# Optimizing a community-based intervention to improve help-seeking for depression care: study protocol for a randomized factorial trial

**DOI:** 10.1186/s13063-025-09014-2

**Published:** 2025-08-16

**Authors:** Nagendra P. Luitel, Brandon A. Kohrt, Bishnu Lamichhane, Anvita Bhardwaj, Kamal Gautam, Mark JD Jordans

**Affiliations:** 1 Research Department, Transcultural Psychosocial Organization (TPO) Nepal, Baluwatar, Kathmandu, Nepal; 2 Center for Global Mental Health Equity, Department of Psychiatry, The George Washington University, Washington, DC USA; 3 Department of Psychiatry, Massachusetts General Hospital, Boston, MA USA; 4 Department of Psychiatry, Harvard Medical School, Boston, MA USA; 5 Department of Pscyhological and Brain Sciences, Boston University, Boston, MA USA; 6 Health Service and Population Research Department, Institute of Psychiatry, Psychology and Neuroscience, Centre for Global Mental Health, King’s College London, London, UK

**Keywords:** Help-seeking, Depression, Multiphase optimization strategy, Nepal, Community intervention

## Abstract

**Background:**

Depression is a common mental health issue that can be effectively managed in primary and community health care settings. However, there is a significant gap between the number of individuals in need of care and those who actually receive treatment, with the greatest gap in low- and middle-income countries (LMICs). Although efforts have been made in LMICs to improve access to mental health services by addressing supply-side barriers, there has been less attention to demand-side obstacles. On the demand side, factors such as stigma, negative attitudes, and limited awareness of available services lead to underutilization of mental health services. This protocol describes a study of active ingredients of a community-based intervention aimed at enhancing help-seeking for depression care in Nepal, a LMIC with low rates of mental health treatment seeking.

**Methods:**

The study will take place in two municipalities in eastern Nepal, utilizing the Multiphase Optimization Strategy (MOST) with a 2 × 3 factorial randomized controlled trial design. Female Community Health Volunteers will be trained to identify individuals with depression using the Community Informant Detection Tool, a proven community-based strategy for proactive case detection, and subsequently implement the Gain Life intervention, which aims to promote help-seeking for depression care. The Gain Life intervention comprises four components: (i) information about depression, (ii) awareness of available services, (iii) stigma reduction by dispelling myths and facts about depression, and (iv) a life transformation story. The study will target the adult population, with eligibility criteria including being 18 years or older, residing in specific municipalities, meeting the CIDT threshold for depression, providing consent, and having proficiency in Nepali. The sample size will be 288, with the primary outcome being help-seeking behaviour.

**Discussion:**

In this protocol paper, we outline how the MOST framework can optimize a community-based intervention aimed at improving help-seeking for depression care. The findings from this study will guide decisions on whether to proceed with a fully randomized controlled trial or conduct an additional optimization study to finalize the intervention components.

**Trial registration:**

ClinicalTrials.gov NCT06574074. Registered on 27 August 2024.

## Introduction

Depression is a prevalent mental health condition that imposes significant emotional and financial burden on individuals, families, and communities [22, 25, 35, 56]. It is estimated that over 322 million people globally are affected by depression [76], accounting for 40.5% of disability-adjusted life years (DALYs) caused by mental illness [74]. It is associated with increased risk of suicide, type-2 diabetes, and heart disease [4, 38, 64] with over 50% of suicide victims having depression [64]. Women are reported to be 2 to 3 times more likely to experience depression than men [51, 73], with a higher risk during the perinatal period, from pregnancy to one year post-delivery [2, 23, 58, 73].

Depression is a treatable and potentially preventable condition [3]. Timely and appropriate help-seeking is crucial for early detection, treatment, and recovery [16]. Research has shown that depression and other mental health conditions can be effectively treated in primary and community health care systems through a task-sharing approach [7, 34, 37]. Despite the availability of evidence-based cost-effective interventions for depression, there is a significant gap between the number of individuals in need of care and those who receive treatment [8, 9, 28, 34, 63, 70, 75]. Studies have reported that 86.3% of people with anxiety, mood, or substance disorders in lower-middle-income countries received no treatment [20]. Additionally, individuals with depression often prefer seeking help from informal sources such as traditional healers, friends, or family members rather than professionals like doctors, psychiatrists, or psychologists [65, 66].

Nepal has made significant strides in improving access to mental health care. Initiatives include translating and adapting the WHO mental health gap action programme intervention guide (mhGAP-IG) for use in Nepal [46], developing a national mental health strategy and action plan [57], creating mental health training manuals for health care providers [41, 44], providing free psychotropic medicines at the lowest level of health care facilities [71], and establishing a standard treatment protocol [43]. The World Health Organization (WHO) has been supporting the government in implementing and scaling up mental health services throughout the country through its special initiatives [55]. Despite these efforts, a large proportion of people (77%) with mental health conditions in Nepal do not receive any treatment [62]. The main reasons for not seeking mental health services are related to individuals’ attitudes and intentions towards mental health problems. For example, the national mental health survey revealed that approximately half of the participants who did not seek care believed that the problem would resolve on its own (48%), followed by a willingness to solve the problem independently (47%), and the perception that they did not have mental health issues (46%) [62].

The underutilization of mental health services in many low- and middle-income countries (LMICs) can be influenced by various factors, including structural and attitudinal barriers. Efforts have been made to improve the availability of mental health services by addressing supply-side barriers through a task-sharing approach and reducing structural barriers. However, demand-side barriers, such as low perceived needs, stigma, and negative attitudes towards mental health treatment, are often overlooked [1]. Stigma and negative attitudes towards mental health care significantly contribute to the underutilization of services and are crucial barriers to seeking help [12, 30, 59, 68]. Internalized stigma is linked to reduced help-seeking behaviour or negatively affects help-seeking [10, 11]. Poor mental health literacy, lack of knowledge about available services, lack of trust in services, cultural practices and beliefs, and low detection within the family are other significant barriers to accessing mental health services [1, 5, 18, 26, 30, 45, 47, 67].

Research has shown a positive relationship between mental health literacy and help-seeking behaviour, indicating that improved mental health literacy can increase the likelihood of seeking help [29, 72]. Similarly, individuals’ attitudes and intentions toward seeking help can predict future behaviour [69], emphasing the importance of interventions that promote positive attitudes toward mental health care to encourage actual help-seeking behaviour [27]. Social-contact-based interventions involving education and personal recovery stories have been used to reduce mental health stigma [50] including in Nepal [40], but they often yield only short-term results [54]. These findings suggest a need for evidence-based interventions to improve help-seeking behaviour for mental health care.

This study aims to develop a community-based intervention to address barriers to seeking help for depression by focusing on improving mental health literacy, increasing awareness of available mental health services, dispelling myths and misconceptions about depression, and changing negative attitudes towards depression care through a recovery story from someone who has experienced depression. The intervention was developed through a systematic review, qualitative study, and input from various stakeholders, including individuals with lived experience and mental health experts. This paper outlines the intervention, the evaluation methods used to assess its delivery strategies, and the testing of its feasibility, acceptability, and appropriateness.

## Methods

### Objective

The aim of this study is to analyze the active components of Gain-Life (Give informAtIoN to transform Life), a community-based intervention designed to promote help-seeking for depression care. The study will examine the effects of intervention components including information on depression, awareness of treatment options, stigma reduction, dispelling myths about depression, and sharing personal transformation experiences, on help-seeking behaviour as the primary outcome and treatment adherence as secondary outcomes. Furthermore, the study will evaluate the delivery methods and assess the feasibility, acceptability, and appropriateness of Gain-Life intervention in encouraging help-seeking for depression care.

1. The primary research question of this study is to determine which component of the Gain-Life intervention has a positive impact on the primary outcome of help-seeking behaviour among individuals with depression. This outcome is measured as the percentage of people seeking care from a health care provider within one month of receiving the intervention.
2. The secondary research question aims to evaluate the intervention’s effects on different outcomes, such as treatment adherence, reduction in depression symptom severity, and the feasibility, acceptability, and appropriateness of Gain-Life in promoting help-seeking for depression treatment.

### Setting

The study will be conducted in two municipalities in the Morang district of eastern Nepal. Morang is one of the most populous districts in the country and also diverse in caste/ethnicity, language, and geography. The total population of Morang district is 1,148,156 according to the 2021 census [61]. The selection of Patharisanischare municipality and Kanepokhari rural municipality for this study was purposeful taking into account factors such as diversity in caste/ethnicity and language, absence of mental health services, lack of FCHVs trained in the community informant detection strategy, and the willingness of local government to provide support. The combined population of Kanepokhari (43,193) and Patharisanischare (72,451) is 115,544 with 54,389 males and 61,255 females [61]. There are 28,643 households, with 10,663 in Kanepokhari and 17,980 in Patharisanischare. The literacy rates in Patharisanischare (81.5%) and Kanepokhari (77.8%) are slightly higher than the national average of 76.2%. Patharisanischare has 10 health facilities (1 municipal level hospital, 2 health posts, and 7 rural health centers), while Kanepokhari has 7 health facilities (1 municipal level hospitals, 2 health posts, and 4 rural health centers). The number of FCHVs in Kanepokhari (*n* = 24) is slightly lower than in Patharisanischare (*n* = 30).

### Study design

This is a single-blinded, individually randomized factorial trial. Behaviour change interventions are complex due to the many interacting components involved [15]. While traditional randomized controlled trial (RCT) designs are considered the gold standard for evaluating the impact of an intervention, they typically do not identify which specific components contribute to desired outcomes [13]. In contrast, factorial design enables the assessment of individual intervention components and their interactions [36]. This approach is appropriate for enhancing the effectiveness, efficiency, and cost-effectiveness of an intervention, ultimately facilitating its scalability to benefit a large population [14].

In this study, we will utilize a Multiphase Optimization Strategy (MOST), an engineering-based approach aimed at effectively optimizing behavioural interventions [13]. MOST comprises three key phases: preparation, optimization, and evaluation. The preparation phase involves developing a conceptual model for the intervention and piloting it to identify “core components” and determine the optimization criteria (e.g. effectiveness, efficiency, cost). The optimization phase utilizes a multifactorial design to conduct a randomized factorial experiment trial using the specific components identified during the preparation phase. Finally, the evaluation phase involves reviewing the trial results and reaching a consensus on the way forward. We will conduct a 2 × 3 factorial individually randomized, single-blinded controlled trial design. Participants detected by Female Community Health Volunteers using the CIDT, will be randomized into one of eight possible combinations of three candidate intervention components ranging from none to all three components (*n* = 36 in each condition). All participants will receive component one, i.e. information about depression. However, the three candidate intervention components, each with two options, include the following: (a) information about available services (on/off), (b) addressing stigma and discrimination (on/off), and (c) life transformation recovery stories (on/off). All four intervention components will be delivered in person on a one-to-one basis.

### Participants and eligibility criteria

The study will be conducted with an adult population. The eligibility criteria for participation in this study include being an adult aged 18 or older residing in Kanepokhari Rural Municipality or Patharisanischare Municipality, those detected through CIDT for depression, providing written consent for participation, and being proficient in speaking and understanding Nepali. Participants receiving mental health services will be excluded from the study. In Nepal, FCHVs, the lowest level of health workers, will receive training to incorporate CIDT into their regular responsibilities. When FCHVs encounter someone in the community matching the narrative, they ask two additional questions about the impact of the condition on daily activities and the individual’s willingness to seek help [31]. If the person meets the criteria and responds positively to at least one of the two questions, FCHVs will schedule the Gain Life intervention.

### Recruitment

Participants will be purposefully recruited through a systematic process. Trained FCHVs will identify potential participants who meet the CIDT criteria, such as individuals whose personal experiences align significantly with the CIDT story, exhibit impaired daily functioning, and express a need for support. Once identified, FCHVs will provide the details (age, gender, location) of individuals who meet CIDT criteria to their supervisor via a phone call. The supervisors will assess the eligibility criteria (i.e. 18 years or older, ability to speak, and CIDT threshold) and send the case details to the first author (NPL) for randomization.

Bi-monthly group meetings will be held for FCHVs to share their progress, discuss challenges encountered during the implementation of the CIDT, and provide motivation. Supervisors will also conduct field visits to offer immediate feedback and enhance FCHVs’ skills in utilizing the CIDT to ensure an effective recruitment strategy.

### Sample sizes and sampling process

The sample size for this study was determined based on the need to detect a difference in help-seeking behaviour between participants receiving the mental health literacy component (i.e. information about depression) alone (40%) and those receiving both the mental health literacy and stigma, myths, and facts about depression components (60%) [32, 53]. With 90% power and a significance level of 0.05, a total sample size of 288 participants is required, with 36 participants per condition. This sample size will allow to determine which component of the Gain-Life intervention positively impacts the primary outcome of help-seeking behaviour among individuals with depression.

### Randomization

Participants will be randomly assigned to one of eight treatment conditions based on four factors: (i) information on depression, (ii) available treatments, (iii) stigma reduction through myths and facts, and (iv) life transformation experiences. Using Microsoft® Excel (Version 16.86), a list of random numbers between one and eight with no duplicates was generated for each FCHV. As the FCHVs enrol participants, the first author assigns the next random number on their list to each participant, indicating the allocated treatment condition. Because each FCHV will recruit between 8 and 10 participants and there are only 8 treatment conditions, some FCHVs ended up delivering each condition at least once, and some conditions will be delivered twice. The table below summarizes the allocation of participants into different intervention components.

The first author will randomize each case using a computer-generated random table and assign them to one of the eight factors presented in Table 1 below. The randomization will be conducted separately for each FCHV to ensure cases are allocated to all 8 factors. The first author will inform the supervisors of the randomization results within 24 h. The supervisors will then inform FCHV which intervention or combination of interventions should be delivered to each case via a phone call.

**Table 1:** Random allocation of participants into different intervention components

Experimental condition/factors	Information about depression	Information about available services	Stigma reduction	Recovery story (video)	Sample size
1	**✓**	**✓**	**✓**	**✓**	36
2	**✓**	**✓**	**✓**		36
3	**✓**	**✓**		**✓**	36
4	**✓**	**✓**			36
5	**✓**		**✓**	**✓**	36
6	**✓**		**✓**		36
7	**✓**			**✓**	36
8	**✓**				36

### Blinding

**Fig. 1:**
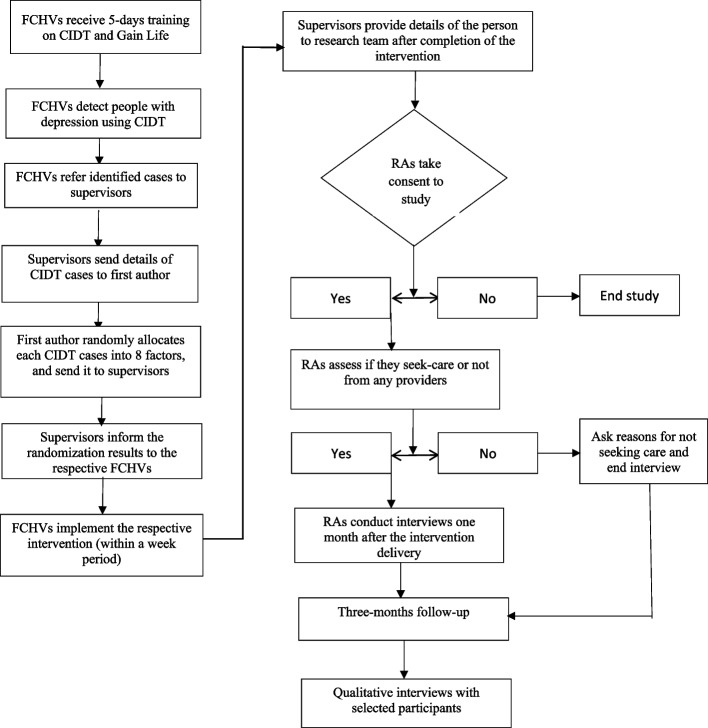
Recruitment process map

Data collectors conducting baseline and follow-up interviews will remain blinded. The Principal Investigator (PI), trainers/supervisors, FCHVs, and participants will not be blinded to the treatment conditions. The PI, who is responsible for managing the study, randomizing participants, and supervising field activities including quality control, will not be blinded to the treatment condition. The statistician, who analyzes the quantitative data for the study, remains blinded (Fig. 1).

#### Assessing and addressing harms

While this study carries a low risk, discussing mental health issues may be distressing for some participants. To mitigate these risks, we will provide access to trained counselors who can effectively handle such situations and make referrals to health care facilities if needed. Participants who report self-harm or suicidal thoughts will be promptly referred to counselors for immediate support.

### Description of the intervention

Previous studies have shown that mental health literacy is associated with help-seeking behaviour, suggesting that improved mental health literacy can increase the likelihood of seeking help [29, 72]. Similarly, several studies have found that higher levels of stigma, particularly related to seeking help for mental health issues and personal stigma, are significantly linked to reduced active help-seeking behaviour [21, 68]. A formative study identified lack of information about available services, lack of knowledge about mental health problems, and lack of support from family members as major barriers to seek care for mental health issues. Therefore, the Gain Life intervention was developed based on the evidence from previous studies and the formative study results. The intervention comprises four components: mental health literacy, stigma reduction, information on available services, and a video featuring a service user’s recovery story. Workshops were conducted with psychologists, psychosocial counsellors, and individuals implementing mental health interventions in the community to develop and finalize the contents for these components. The draft intervention was pilot tested with ten FCHVs in a different district to gather their feedback on the content, delivery mechanism, training duration and methods, and supervision needs.

#### Information about depression

The aim of this component is to raise awareness about depression, with a particular focus on its causes, manifestations, and the potential negative consequences of untreated depression. Previous studies have indicated that the causes of depression are often linked to stressful life events, socio-cultural factors, and genetic predispositions, rather than solely biological factors. In this component, we have highlighted various contextual factors, including cultural practices and beliefs, stressful life events such as natural disasters (e.g. earthquakes, landslides), economic hardships, changes in family structures, family stress, educational stress, genetic influences, social discrimination and exclusion, substance abuse, and chronic illnesses like cancer and HIV/AIDS.

In addition to the common symptoms of depression outlined in standard guidelines, we have included culturally and context-specific symptoms frequently reported by individuals with depression in Nepal, such as stomach-ache, headaches, unexplained body pains, and tingling sensations. Research has shown that individuals are more likely to seek care for their illness if they understand the negative consequences of leaving it untreated. Therefore, we have included information on the potential consequences of untreated depression, which can range from difficulties in daily activities and increased chronicity of the illness to reduced treatment effectiveness and higher treatment costs. This component is designed as a flipchart with animated images to engage participants. FCHVs can use the flipchart on a table or in a planned space, turning the pages to display images on participants’ side and the page containing the content on their side.

#### Information about available services

The second component of the intervention provides detailed information about mental health services available in the community, district, and neighbouring districts. This includes the types of services offered, costs, distance to service providers, and operating hours. Lack of knowledge about available services has been identified as a barrier to seeking mental health care. Studies have shown that patients often do not know where to seek help and may resort to traditional healers or avoid seeking care altogether. However, mental health services are now accessible in primary and community health care facilities through a task-sharing approach. The aim of this intervention component is to address the lack of awareness about available services and assist individuals in identifying suitable sources of support. It encompasses all treatment options, including primary health care providers who are on the mental health Gap Action Program (mhGAP) intervention guide and municipal hospitals, as well as specialist services within the district and neighbouring districts. This component is being produced as a booklet with images of specific health facilities and their surroundings on one side, and descriptions of available services and services that are not available on the other side. This format helps FCHVs associate the images with available services while implementing the component.

#### Stigma, myths and facts about depression

The purpose of this component is to address stigma and misconceptions surrounding depression by addressing the root causes of stigma, dispelling myths, and providing accurate information about depression. The component covers the stigma surrounding mental health issues, common stigmatized behaviours experienced by individuals with depression, and how stigma and discrimination can hinder help-seeking behaviour. The second part of the component focuses on debunking prevalent myths about depression and presenting the facts. This information is presented in a table format, with one column listing myths and another column providing factual information. The component is designed as a wall calendar with animated images to enhance understanding, particularly for participants with reading difficulties. FCHVs can use the calendar to display information on a wall or facilitate discussions by connecting the text with corresponding images.

#### Recovery stories of persons with lived experience

The final component of the Gain Life intervention showcases recovery stories of individuals who have triumphed over depression. This component comprises two 8-min video stories featuring individuals who successfully recovered from depression. The first video narrates the story of a woman from Parbat, a district in mid-western Nepal, who overcomes depression by seeking treatment from various sources and receiving ongoing care from primary health care facilities near her home. She was a person with lived experience trained in PhotoVoice to create a recovery narrative [44]. She also received support from psychosocial counsellors and her family. The second story is about a young man who battled depression and suicidal thoughts, surviving a suicide attempt and eventually opening up to his family, especially his parents, about his struggles. The video demonstrates how this young man overcame depression with the help of his parents, mental health professionals, and his own determination. FCHVs show the videos on a tablet and discuss them briefly with each participant. They focus on the individuals’ problems, symptoms, types of services received, and the crucial role of family members in the treatment process and recovery.

### Training

#### Training to FCHVs

FCHVs will receive a 5-day training that includes the Community Informant Detection Tool (CIDT) and the Gain Life intervention. The first part of the training will cover basic concepts of mental health, reasons for underutilization of mental health services, and the CIDT’s purpose of identifying individuals with depression. FCHVs will learn to use vignettes for detection, recognize symptoms, and assess the need for depression support. Role-playing exercises will emphasize seeking help and involving family members.

The second part of the training will focus on the Gain Life intervention, covering theoretical and practical aspects. FCHVs will learn about mental health problems, depression, causes, symptoms, consequences of untreated depression, available mental health services, stigma associated with mental health problems, and strategies to reduce stigma. Role-playing activities in small groups will help FCHVs gain confidence in implementing the intervention effectively.

#### Training to primary health care providers

Primary health care workers in Kanepokhari and Pathari-sanischare municipalities have not received training on mental health. Therefore, they will undergo a 5-day mhGAP training based on the module developed by the government of Nepal. The training will focus on the assessment, diagnosis, and management of mental health conditions such as depression, psychosis, alcohol use disorder, epilepsy, anxiety, and child and adolescent mental and behavioural problems. The training will be conducted by a psychiatrist and a psychologist. Female Community Health Volunteers (FCHVs) who receive training in the Gain Life intervention will provide information on the services available in the health facilities where health workers are trained on mhGAP.

### Implementation process of Gain Life intervention

FCHVs will utilize the CIDT in their daily tasks to identify individuals experiencing depression. They will initially consider individuals whose experiences resonate with the CIDT vignette. Upon identifying a potential candidate, FCHVs will engage in a consultation to evaluate the impact of the issue on the individual’s daily life and their readiness to seek assistance. If the individual agrees to participate in the Gain Life intervention, FCHVs will arrange a suitable date and time for its implementation.

As per the study protocol, participants may receive all four components of the Gain Life intervention or a combination of three, two, or one component. FCHVs will coordinate the intervention implementation accordingly, with a maximum of two components being delivered simultaneously. In cases where three components are required, they will be administered on separate days. The intervention sessions will take place in confidential settings, such as the participant’s or FCHV’s residence, to ensure privacy. While family members are encouraged to join, participants who prefer not to involve them can opt for individual sessions.

Participants may choose not to receive all allocated components of the intervention for reasons such as feeling that they do not need the additional components, starting treatment after receiving initial components, or other contextual factors. These participants will still be included in the analysis using an intention-to-treat approach. Additionally, there will be no restrictions on participants receiving other interventions, support, or attending awareness programmes. These aspects will be explored in the qualitative component of the intervention.

### Supervision of FCHVs

Trained FCHVs will receive regular supervision from psychosocial counsellors in three ways. Firstly, they will participate in group supervision sessions twice a month to discuss challenges encountered while implementing CIDT and Gain Life interventions. The psychosocial counsellor will offer practical guidance and skills to address these challenges. Group supervision sessions will be held separately for FCHVs in Kanepokhari Rural Municipality and Patharisanischare Municipality. Secondly, each FCHV will undergo two live supervisions per month, during which the counselor will observe a Gain Life session. These sessions will only be observed if the participant or their family member feels comfortable. Lastly, the psychosocial counsellors will contact each FCHV before implementing the intervention to ensure they are using the correct components as allocated randomly.

## Outcome measures

### Primary outcome

#### Help-seeking behaviour

Changes in help-seeking behaviour will be assessed by determining if participants sought mental health treatment within one month after attending the FCHVs intervention. Mental health treatment is defined as treatment provided by health care providers including trained primary health care workers, doctors, and mental health specialists. This does not include treatment provided by traditional healers and religious leaders. Research assistants, who are unaware of the participants’ assignment to the intervention, will visit their homes one month post-intervention. They will inquire about the participants’ interactions with FCHVs, the places/providers they visited for seeking care, and the reasons for choosing a specific provider using a semi-structured questionnaire. We will not verify the accuracy of the treatment they received. Participants who did not seek treatment will also be asked about the reasons for not seeking care and their plans to seek treatment soon. Participants will be asked a “yes” or “no” question: “Did you seek help for your health issue after attending the FCHV session?” with a yes or no response. We will report the percentage of participants who respond “yes” to this question as an indicator of seeking treatment.

### Secondary outcome

#### Treatment adherence

Treatment adherence will be monitored by following up with participants for three months after they start treatment. The three-month follow-up period was chosen based on feasibility and previous studies [17, 33]. Participants who begin treatment one month after the FCHVs intervention will be followed up after three months to evaluate their ongoing care. During the follow-up, participants will be asked if they are still receiving care from the same providers, if they have switched providers, or if they have stopped treatment. Participants who switched providers will be asked about the reasons for the change, while those who discontinued treatment will be asked about the reasons for their decision to stop receiving services. Participants will be asked “yes” or “no” questions: “Have you been receiving treatment for your health issues since we last met three months ago?” We will report the percentage of participants who respond “yes” to this question as an indicator of treatment adherence.

### Mediators

We will examine the mediating effects on help-seeking behaviour at the three-month follow-up. The mediating variables include knowledge, attitude, and perception of depression, as well as the severity of depressive symptoms. These mediating variables were selected based on the impact of these knowledge, attitude, and perception of depression on help-seeking [10, 11, 29, 69].

### Knowledge, attitudes, perceptions of depression and available services

Participants’ knowledge, attitudes, and perceptions regarding depression and available services will be assessed during a three-month follow-up. Three structured questionnaires, each with four response options, have been developed based on the intervention content. The knowledge about depression scale consists of 7 items, the stigma and myths scale consists of 5 items, and the knowledge about available services scale consists of 8 items. These scales will be asked during the three-month follow-up, not at baseline. Each question will have one correct response and three incorrect responses. Participants will choose the correct response for each question after a research assistant reads the question and all possible responses. Composite scores will be calculated based on the number of correct responses for each scale for analysis. We will report the number of correct responses in each domain: knowledge, attitudes, and perceptions on depression. Additionally, we will also use continuous scores (i.e. total score of each domain) when performing mediator analysis.

### Symptom severity

The severity of depression symptoms will be assessed using the locally validated Patient Health Questionnaire (PHQ-9) three months after treatment initiation. The PHQ-9 is a nine-item tool commonly used to evaluate depression symptoms. Participants will rate nine common symptoms of depression based on their experiences over the previous two weeks using a 4-point scale ranging from 0 (not at all) to 3 (always) [24]. The PHQ-9 has been translated, culturally adapted, and validated in Nepal with a sensitivity of 0.94 and specificity of 0.80 for the recommended cut-off score of ≥ 10 for screening individuals with depression [42]. We will use the total scores of the PHQ-9, which range from 0 to 27, to examine the mediating effects of depression symptom severity in individuals seeking help. A higher score on the PHQ-9 indicates a more severe level of depression [24].

### Implementation measures

Data on fidelity, feasibility, acceptability, and appropriateness of the intervention delivered by FCHVs in community settings will be collected for each intervention component and delivery strategy.

### Fidelity of the implementation of Gain Life

Fidelity in implementing the components and combination of components in the Gain Life intervention will be assessed by two supervisors. They will observe sessions and engage with Female Community Health Volunteers (FCHVs) within 24 h of the intervention. A structured fidelity checklist will be utilized to evaluate the skills, content, and delivery methods of each intervention component. The checklist is based on the content and delivery methods outlined in different components of the intervention. It will ascertain if FCHVs covered all the methods and content in a particular component, and if they incorporated content from other components not included in that session. Supervisors will observe at least two sessions of each FCHV, and two fidelity checklists will be completed through discussions with the FCHVs within 24 h of the intervention.

### Feasibility, acceptability and appropriateness

The feasibility, acceptability, and appropriateness of the intervention will be evaluated using qualitative methods, including individual interviews (IDIs) and focus group discussions (FGDs). FGDs will involve Female Community Health Volunteers (FCHVs) who implemented the intervention to assess their experience with using the CIDT to identify individuals with depression and implementing the Gain Life intervention. Each focus group will include six to eight FCHVs.

Individual interviews will be conducted with three participant groups: those who did not receive treatment, those who started but discontinued treatment, and those who are still receiving care three months after participating in the Gain Life intervention. Some interviews will also involve family members who attended the intervention. Interviews with participants who did not start the intervention will take place two months after their participation, while interviews with the other two groups (those who dropped out and those still receiving care) will occur after a three-month follow-up period.

### Process evaluation

The implementation process, including training and supervision of FCHVs, as well as barriers and facilitators of the intervention, will be documented by a separate individual through observation of training and supervision sessions and interviews with supervisors.

### Data management and analysis

Quantitative data will be collected using an Android tablet with questionnaire applications. The data will be stored in the TPO Nepal server. Data collected on the ODK platform will be transferred to the TPO Nepal server, and any inconsistencies in data collection will be monitored regularly by the research supervisor. Inconsistencies and errors in the data will be minimized manually.

### Quantitative data analysis

Quantitative data analysis will be conducted using StataIC 15.1 and MPlus (v8) [60]. Baseline characteristics of the participants (such as age, sex, caste/ethnicity, education) will be compared to assess balanced randomization. An intent-to-treat model will be used. Participants who do not receive all allocated intervention components for various reasons will be included in the intent-to-treat analysis. Despite the short three-month interval between the baseline and follow-up interviews, there is a risk of participant dropout during the follow-up period, possibly due to work-related mobility. To address this issue, we also plan to conduct the follow-up interviews via phone to minimize the risk of dropout. Any missing data or loss to follow-up will be carefully examined, and if necessary, we will utilize multiple imputation techniques to handle missing data effectively.

### Primary outcome

As recommended for factorial designs, effect coding will be utilized for all analyses [13]. The primary outcome of interest is change in help-seeking behaviour. This will be evaluated by determining the proportion of participants who attended the Gain Life intervention and sought care from health care providers rather than traditional or religious leaders. This will be measured by the number of participants who received the Gain Life intervention and sought treatment from health care providers. General linear regression models will be used to test the main effects of the Gain Life intervention components and their association with the primary outcome of interest [52].

### Secondary outcomes

Our secondary outcome is treatment adherence for depression care. This will be determined based on the number of participants who initiate treatment one-month post-intervention and continue to engage in care during the three-month follow-up period. General linear regression models will be used to test the secondary outcomes as well.

### Mediators

Previous studies have demonstrated a positive correlation between mental health literacy, attitudes, stigma, and symptom severity in relation to help-seeking behaviour [10, 11, 29, 69]. Our formative study also indicated that individuals who were familiar with depression symptoms and aware of mental health service providers and locations were more inclined to seek help from health care providers. Therefore, we aim to investigate whether participants’ knowledge, attitudes, and stigma can act as mediators in influencing help-seeking behaviour. Furthermore, we will also assess the symptom severity of depression, as measured by the PHQ-9 at a three-month follow-up, to determine if it plays a mediating role in help-seeking behaviour. We will use the Baron and Kenny method along with a path analysis to examine the mediation effects. For the Baron and Kenny method, a series of general linear regression models will be tested to assess mediation [49]. Mplus will be used to conduct a path analysis looking at the direct and indirect effects to determine if the hypothesized mediator is in fact a mediator in the relationship between the predictor variable and outcome of interest [19].

### Moderators

We will assess how socio-demographic factors such as caste/ethnicity, age, gender, education level, occupation, and religion moderate the intervention’s impact through general linear regression models and interaction variables. Previous studies in Nepal have shown that gender, caste/ethnicity, and occupation can affect rates of depression and anxiety [39, 48]. These analyses are crucial for promoting equitable access to mental health care in Nepal.

### Qualitative data analysis

Each interview or focus group discussion will be recorded and transcribed in Nepali immediately after the fieldwork. The transcripts will then be translated into English for analysis by a skilled translator. Two members of the research team will independently code 10% of the interviews or focus group discussions following the CFIR guidelines. A review of all the codes for each interview will be carried out until the research team members reach a consensus on the codes to be applied to specific text segments. Once consensus is reached, interview transcripts will be entered, coded, and analyzed in NVIVO.

### Dissemination

We will share the results with key stakeholders in Kanepokhari Rural Municipality and Patharisanischare Municipality. Additionally, we will organize a dissemination programme in Kathmandu for stakeholders involved in mental health, including senior officers from the Ministry of Health and Population, representatives from non-governmental organizations, international non-governmental organizations, and other civil society organizations. The study findings will also be published in academic journals.

### Timeline

**Fig. 2:**
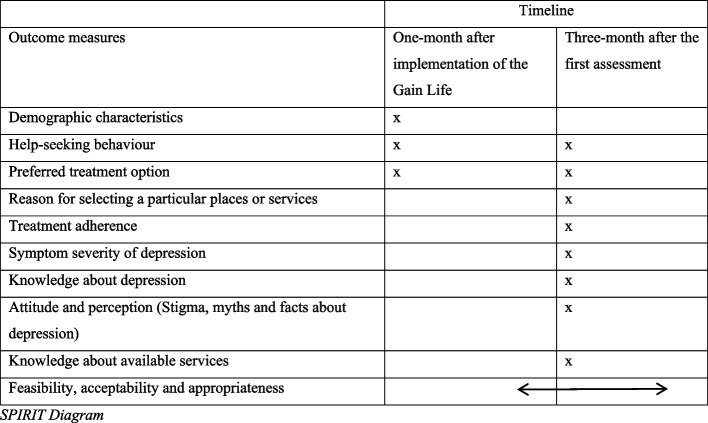
Schedule of enrolment, interventions, and assessments

The training of FCHVs on CIDT and on Gain Life intervention began in May. They began implementing CIDT and the intervention in June. Data collection for the baseline survey commenced in July 2024. The enrollment and assessment schedule, following the SPIRIT guidelines, is outlined in Fig. 2 below. The protocol was developed in strict adherence to the Standard Protocol Items: Recommendations for Intervention Trials 2013 (SPIRIT) guidelines [6].

## Discussion

In this study, we utilized the MOST framework to optimize a community-based intervention aimed at increasing help-seeking for depression care. Through a factorial design, our goal is to develop an effective intervention that raises awareness about depression, available services, and reduces stigma. The study will examine the impact and interaction of intervention components such as depression information, treatment awareness, dispelling myths, and life transformation experiences. The primary outcome will be changes in help-seeking behaviour, with treatment adherence as a secondary outcome.

The strengths of our study include the following: (i) the use of multiple methods and stakeholders in the development of the intervention, such as a systematic review, formative study, consultative workshops with various stakeholders, and pilot testing with a group of 10 FCHVs in another district; (ii) while most global mental health research focuses on developing and evaluating interventions to address supply-side barriers, this study targets demand-side barriers by using the innovative study design MOST, enabling us to evaluate the effectiveness of intervention components through an iterative optimization process; and (iii) the implementation of the intervention and study in multicultural, multilingual, and multi-ethnic communities in eastern Nepal, allowing for generalization of results throughout Nepal.

We anticipate challenges in maintaining intervention fidelity, as participants may seek additional information from FCHVs beyond what is included in the intervention packages or in a particular component. Additionally, ensuring fidelity in delivering the core components and the combination of intervention packages by the same FCHVs may be challenging. To address this, fidelity checklists will be used to guide delivery, and regular supervision sessions will be conducted to support FCHVs in maintaining fidelity.

The study has some limitations, including being single-blinded, with only RAs and the statistician blinded to the study allocation. We have included four intervention components, including information about depression as the core component and three candidate components, due to resource constraints. There may be other factors influencing help-seeking for depression that were not included in this study. The results will guide decisions on progressing to a fully powered randomized controlled trial and further optimization with new intervention components.

## Data Availability

Data sharing is not applicable to this article as no datasets were generated or analyzed in the current study.

## References

[1] Andrade LH, Alonso J, Mneimneh Z, Wells JE, Al-Hamzawi A, Borges G, Bromet E, Bruffaerts R, de Girolamo G, de Graaf R, Florescu S, Gureje O, Hinkov HR, Hu C, Huang Y, Hwang I, Jin R, Karam EG, Kovess-Masfety V, Levinson D, Matschinger H, O’Neill S, Posada-Villa J, Sagar R, Sampson NA, Sasu C, Stein DJ, Takeshima T, Viana MC, Xavier M, Kessler RC. Barriers to mental health treatment: results from the WHO world mental health surveys. Psychol Med. 2014;44(6):1303–17. 10.1017/s0033291713001943. 23931656 10.1017/S0033291713001943 PMC4100460

[2] Austin MP. Antenatal screening and early intervention for “perinatal” distress, depression and anxiety: where to from here? Arch Womens Ment Health. 2004;7(1):1–6. 10.1007/s00737-003-0034-4. 14963727 10.1007/s00737-003-0034-4

[3] Barrera AZ, Torres LD, Munoz RF. Prevention of depression: the state of the science at the beginning of the 21st century. Int Rev Psychiatry. 2007;19(6):655–70. 10.1080/09540260701797894. 18092243 10.1080/09540260701797894

[4] Baxter AJ, Charlson FJ, Somerville AJ, Whiteford HA. Mental disorders as risk factors: assessing the evidence for the global burden of disease study. BMC Med. 2011;9:134. 10.1186/1741-7015-9-134. 22176705 10.1186/1741-7015-9-134 PMC3305628

[5] Brenman NF, Luitel NP, Mall S, Jordans MJD. Demand and access to mental health services: a qualitative formative study in Nepal. BMC Int Health Hum Rights. 2014;14:22. 10.1186/1472-698x-14-22. 25084826 10.1186/1472-698X-14-22 PMC4126616

[6] Chan AW, Tetzlaff JM, Altman DG, Laupacis A, Gøtzsche PC, Krleža-Jerić K, Hróbjartsson A, Mann H, Dickersin K, Berlin JA, Doré CJ, Parulekar WR, Summerskill WSM, Groves T, Schulz KF, Sox HC, Rockhold FW, Rennie D, Moher D. SPIRIT 2013 statement: defining standard protocol items for clinical trials. Ann Intern Med. 2013;158(3):200–7. 10.7326/0003-4819-158-3-201302050-00583. 23295957 10.7326/0003-4819-158-3-201302050-00583 PMC5114123

[7] Chaulagain A, Pacione L, Abdulmalik J, Hughes P, Oksana K, Chumak S, Mendoza J, Avetisyan K, Ghazaryan G, Gasparyan K, Chkonia E, Servili C, Chowdhury N, Pinchuk I, Belfar M, Guerrero A, Panteleeva L, Skokauskas N. WHO mental health gap action programme intervention guide (mhGAP-IG): the first pre-service training study. Int J Ment Health Syst. 2020;14: 47. 10.1186/s13033-020-00379-2. 32612675 10.1186/s13033-020-00379-2 PMC7325034

[8] Chowdhary N, Anand A, Dimidjian S, Shinde S, Weobong B, Balaji M, Patel V. The Healthy Activity Program lay counsellor delivered treatment for severe depression in India: systematic development and randomised evaluation. Br J Psychiatry. 2015. 10.1192/bjp.bp.114.161075 10.1192/bjp.bp.114.161075 PMC4816974 26494875

[9] Christensen H, Griffiths KM, Gulliver A, Clack D, Kljakovic M, Wells L. Models in the delivery of depression care: a systematic review of randomised and controlled intervention trials. BMC Fam Pract. 2008;9:25. 10.1186/1471-2296-9-25. 18454878 10.1186/1471-2296-9-25 PMC2390560

[10] Clement S, Schauman O, Graham T, Maggioni F, Evans-Lacko S, Bezborodovs N, Morgan C, Rüsch N, Brown JSL, Thornicroft G. What is the impact of mental health-related stigma on help-seeking? A systematic review of quantitative and qualitative studies. Psychol Med. 2015;45(1):11–27. 10.1017/s0033291714000129. 24569086 10.1017/S0033291714000129

[11] Clement S, Williams P, Farrelly S, Hatch SL, Schauman O, Jeffery D, Henderson RC, Thornicroft G. Mental health-related discrimination as a predictor of low engagement with mental health services. Psychiatr Serv. 2015;66(2):171–6. 10.1176/appi.ps.201300448. 25642612 10.1176/appi.ps.201300448

[12] Codony M, Alonso J, Almansa J, Bernert S, de Girolamo G, de Graaf R, Kessler RC. Perceived need for mental health care and service use among adults in Western Europe: results of the ESEMeD project. Psychiatr Serv. 2009;60(8):1051–8. 10.1176/appi.ps.60.8.1051. 19648192 10.1176/ps.2009.60.8.1051

[13] Collins LM. Optimization of behavioral, biobehavioral, and biomedical interventions. The Multiphase Optimization Strategy (MOST). 2018. Cham, Switzerland: Springer.

[14] Collins LM, Baker TB, Mermelstein RJ, Piper ME, Jorenby DE, Smith SS, Christiansen BA, Schlam TR, Cook JW, Fiore MC. The multiphase optimization strategy for engineering effective tobacco use interventions. Ann Behav Med. 2011;41(2):208–26. 10.1007/s12160-010-9253-x. 21132416 10.1007/s12160-010-9253-x PMC3053423

[15] Craig P, Dieppe P, Macintyre S, Michie S, Nazareth I, Petticrew M. Developing and evaluating complex interventions: the new Medical Research Council guidance. Int J Nurs Stud. 2013;50(5):587–92. 10.1016/j.ijnurstu.2012.09.010. 23159157 10.1016/j.ijnurstu.2012.09.010

[16] Dawson DA, Grant BF, Stinson FS, Chou PS. Estimating the effect of help-seeking on achieving recovery from alcohol dependence. Addiction. 2006;101(6):824–34. 10.1111/j.1360-0443.2006.01433.x. 16696626 10.1111/j.1360-0443.2006.01433.x

[17] Del Pino-Sedeño T, González-Pacheco H, González de León B, Serrano-Pérez P, Acosta Artiles FJ, Valcarcel-Nazco C, Trujillo-Martín MM. Effectiveness of interventions to improve adherence to antidepressant medication in patients with depressive disorders: a cluster randomized controlled trial. Front Pub Health. 2024;12:1320159. 10.3389/fpubh.2024.1320159. 38633230 10.3389/fpubh.2024.1320159 PMC11022850

[18] Devkota G, Basnet P, Thapa B, Subedi M. Factors affecting utilization of mental health services from primary health care (PHC) facilities of western hilly district of Nepal. PLoS ONE. 2021;16(4): e0250694. 33930894 10.1371/journal.pone.0250694 PMC8087454

[19] Edwards JR, Lambert LS. Methods for integrating moderation and mediation: a general analytical framework using moderated path analysis. Psychol Methods. 2007;12(1):1–22. 10.1037/1082-989x.12.1.1. 17402809 10.1037/1082-989X.12.1.1

[20] Evans-Lacko S, Aguilar-Gaxiola S, Al-Hamzawi A, Alonso J, Benjet C, Bruffaerts R, Chiu WT, Florescu S, de Girolamo G, Gureje O, Haro JM, He Y, Hu C, Karam EG, Kawakami N, Lee S, Lund C, Kovess-Masfety V, Levinson D, Navarro-Mateu F, Pennell BE, Sampson NA, Scott KM, Tachimori H, ten Have M, Viana MC, Williams DR, Wojtyniak BJ, Zarkov Z, Kessler RC, Chatterji S, Thornicroft G. Socio-economic variations in the mental health treatment gap for people with anxiety, mood, and substance use disorders: results from the WHO world mental health (WMH) surveys. Psychol Med. 2018;48(9):1560–71. 10.1017/s0033291717003336. 29173244 10.1017/S0033291717003336 PMC6878971

[21] Evans L, Chang A, Dehon J, Streb M, Bruce M, Clark E, Handal P. The relationships between perceived mental illness prevalence, mental illness stigma, and attitudes toward help-seeking. Curr Psychol. 2023. 10.1007/s12144-023-04329-2. 37359578 10.1007/s12144-023-04329-2 PMC9975862

[22] Ferrari AJ, Charlson FJ, Norman RE, Patten SB, Freedman G, Murray CJL, Vos T, Whiteford HA. Burden of depressive disorders by country, sex, age, and year: findings from the Global Burden of Disease Study 2010. PLoS Med. 2013;10(11): e1001547. 10.1371/journal.pmed.1001547. 24223526 10.1371/journal.pmed.1001547 PMC3818162

[23] Gavin NI, Bradley NG, Lohr KN, Meltzer-Brody S, Gartlehner G, Swinson T. Perinatal depression: a systematic review of prevalence and incidence. Obstet Gynecol. 2005;106(5, Part 1):1071–83. 16260528 10.1097/01.AOG.0000183597.31630.db

[24] Gilbody S, Richards D, Brealey S, Hewitt C. Screening for depression in medical settings with the patient health questionnaire (PHQ): a diagnostic meta-analysis. J Gen Intern Med. 2007;22(11):1596–602. 10.1007/s11606-007-0333-y. 17874169 10.1007/s11606-007-0333-y PMC2219806

[25] Greenberg PE, Fournier AA, Sisitsky T, Pike CT, Kessler RC. The economic burden of adults with major depressive disorder in the United States (2005 and 2010). J Clin Psychiatry. 2015;76(2):155–62. 10.4088/JCP.14m09298. 25742202 10.4088/JCP.14m09298

[26] Gulliver A, Griffiths KM, Christensen H. Perceived barriers and facilitators to mental health help-seeking in young people: a systematic review. BMC Psychiatry. 2010;10: 113. 10.1186/1471-244x-10-113. 21192795 10.1186/1471-244X-10-113 PMC3022639

[27] Gulliver A, Griffiths KM, Christensen H, Brewer JL. A systematic review of help-seeking interventions for depression, anxiety and general psychological distress. BMC Psychiatry. 2012;12: 81. 10.1186/1471-244x-12-81. 22799879 10.1186/1471-244X-12-81 PMC3464688

[28] Holvast F, Massoudi B, Oude Voshaar RC, Verhaak PFM. Non-pharmacological treatment for depressed older patients in primary care: a systematic review and meta-analysis. PLoS ONE. 2017;12(9): e0184666. 10.1371/journal.pone.0184666. 28938015 10.1371/journal.pone.0184666 PMC5609744

[29] Iswanto ED, Ayubi D. The relationship of mental health literacy to help seeking behavior: systematic review. J Soc Res. 2023. 10.55324/josr.v2i3.726.

[30] Jack-Ide IO, Uys L. Barriers to mental health services utilization in the Niger Delta region of Nigeria: service users’ perspectives. Pan Afr Med J. 2013;14:159. 10.11604/pamj.2013.14.159.1970. 23785564 10.11604/pamj.2013.14.159.1970 PMC3683509

[31] Jordans M JD, Kohrt BA, Luitel NP, Komproe IH, Lund C. Accuracy of proactive case finding for mental disorders by community informants in Nepal. Br J Psychiatry. 2015;207(6):501–6. 10.1192/bjp.bp.113.141077 PMC4664856 26450582

[32] Jordans MJD, Kohrt BA, Luitel NP, Lund C, Komproe IH. Proactive community case-finding to facilitate treatment seeking for mental disorders, Nepal. Bull World Health Organ. 2017;95(7):531–6. 10.2471/blt.16.189282. 28670018 10.2471/BLT.16.189282 PMC5487974

[33] Jordans MJD, Kohrt BA, Sangraula M, Turner EL, Wang X, Shrestha P, Ghimire R, van’t Hof E, Bryant RA, Dawson KS, Marahatta K, Luitel NP, van Ommeren M. Effectiveness of group problem management plus, a brief psychological intervention for adults affected by humanitarian disasters in Nepal: a cluster randomized controlled trial. PLoS Med. 2021;18(6):e1003621. 10.1371/journal.pmed.1003621. 34138875 10.1371/journal.pmed.1003621 PMC8211182

[34] Jordans MJD, Luitel NP, Kohrt BA, Rathod SD, Garman EC, De Silva M, Komproe IH, Patel V, Lund C. Community-, facility-, and individual-level outcomes of a district mental healthcare plan in a low-resource setting in Nepal: a population-based evaluation. PLoS Med. 2019;16(2): e1002748. 10.1371/journal.pmed.1002748. 30763321 10.1371/journal.pmed.1002748 PMC6375569

[35] Katzman MA, Anand L, Furtado M, Chokka P. Food for thought: understanding the value, variety and usage of management algorithms for major depressive disorder. Psychiatry Res. 2014;220(Suppl 1):S3-14. 10.1016/s0165-1781(14)70002-2. 25539872 10.1016/S0165-1781(14)70002-2

[36] Kenfield SA, Philip EJ, Phillips SM, Meyerhardt JA, Chan JM, Atreya CE, Kim M-O, Harris Q, Steiding P, Macaire G, McCullough ML, Piawah S, Johnson WY, Kurttila FA, Lewis WL, Pesmen C, Watson Y, Van Blarigan EL. Optimizing intervention tools to improve nutrition and physical activity for colorectal cancer survivors (tools to be fit): study protocol of a randomized factorial experiment. Contemp Clin Trials. 2022;123: 107009. 10.1016/j.cct.2022.107009. 36396066 10.1016/j.cct.2022.107009 PMC10561599

[37] Keynejad R, Spagnolo J, Thornicroft G. WHO mental health gap action programme (mhGAP) intervention guide: updated systematic review on evidence and impact. Evid Based Ment Health. 2021;24(3):124–30. 10.1136/ebmental-2021-300254. 33903119 10.1136/ebmental-2021-300254 PMC8311089

[38] Knol MJ, Twisk JW, Beekman AT, Heine RJ, Snoek FJ, Pouwer F. Depression as a risk factor for the onset of type 2 diabetes mellitus. A meta-analysis. Diabetologia. 2006;49(5):837–45. 10.1007/s00125-006-0159-x. 16520921 10.1007/s00125-006-0159-x

[39] Kohrt BA. Vulnerable social groups in post-conflict settings: a mixed-methods policy analysis and epidemiology study of caste and psychological morbidity in Nepal. Intervention. 2009;7:239–64.

[40] Kohrt BA, Jordans MJD, Turner EL, Rai S, Gurung D, Dhakal M, Bhardwaj A, Lamichhane J, Singla DR, Lund C, Patel V, Luitel NP, Sikkema KJ. Collaboration with people with lived experience of mental illness to reduce stigma and improve primary care services: a pilot cluster randomized clinical trial. JAMA Netw Open. 2021;4(11):e2131475. 10.1001/jamanetworkopen.2021.31475. 34730821 10.1001/jamanetworkopen.2021.31475 PMC8567115

[41] Kohrt BA, Jordans MJD, Turner EL, Sikkema KJ, Luitel NP, Rai S, Singla DR, Lamichhane J, Lund C, Patel V. Reducing stigma among healthcare providers to improve mental health services (RESHAPE): protocol for a pilot cluster randomized controlled trial of a stigma reduction intervention for training primary healthcare workers in Nepal. Pilot Feasibility Stud. 2018;4: 36. 10.1186/s40814-018-0234-3. 29403650 10.1186/s40814-018-0234-3 PMC5781273

[42] Kohrt BA, Luitel NP, Acharya P, Jordans MJD. Detection of depression in low resource settings: validation of the patient health questionnaire (PHQ-9) and cultural concepts of distress in Nepal. BMC Psychiatry. 2016;16:58. 10.1186/s12888-016-0768-y. 26951403 10.1186/s12888-016-0768-y PMC4782581

[43] Kohrt BA, Turner EL, Gurung D, Wang X, Neupane M, Luitel NP, Kartha MR, Poudyal A, Singh R, Rai S, Baral PP, McCutchan S, Gronholm PC, Hanlon C, Lempp H, Lund C, Thornicroft G, Gautam K, Jordans MJD. Implementation strategy in collaboration with people with lived experience of mental illness to reduce stigma among primary care providers in Nepal (RESHAPE): protocol for a type 3 hybrid implementation effectiveness cluster randomized controlled trial. Implement Sci. 2022;17(1): 39. 10.1186/s13012-022-01202-x. 35710491 10.1186/s13012-022-01202-x PMC9205129

[44] Kohrt BA, Turner EL, Rai S, Bhardwaj A, Sikkema KJ, Adelekun A, Dhakal M, Luitel NP, Lund C, Patel V, Jordans MJD. Reducing mental illness stigma in healthcare settings: proof of concept for a social contact intervention to address what matters most for primary care providers. Soc Sci Med. 2020;250: 112852. 10.1016/j.socscimed.2020.112852. 32135459 10.1016/j.socscimed.2020.112852 PMC7429294

[45] Lakshmana G, Sangeetha V, Pandey V. Community perception of accessibility and barriers to utilizing mental health services. J Educ Health Promot. 2022;11:56. 10.4103/jehp.jehp_342_21. 35372598 10.4103/jehp.jehp_342_21 PMC8974990

[46] Luitel NP, Breuer E, Adhikari A, Kohrt BA, Lund C, Komproe IH, Jordans MJD. Process evaluation of a district mental healthcare plan in Nepal: a mixed-methods case study. BJPsych Open. 2020;6(4):e77. 10.1192/bjo.2020.60. 32718381 10.1192/bjo.2020.60 PMC7443901

[47] Luitel NP, Jordans MJD, Kohrt BA, Rathod SD, Komproe IH. Treatment gap and barriers for mental health care: a cross-sectional community survey in Nepal. PLoS ONE. 2017;12(8): e0183223. 10.1371/journal.pone.0183223. 28817734 10.1371/journal.pone.0183223 PMC5560728

[48] Luitel NP, Jordans MJD, Sapkota RP, Tol WA, Kohrt BA, Thapa SB, Komproe IH, Sharma B. Conflict and mental health: a cross-sectional epidemiological study in Nepal. Soc Psychiatry Psychiatr Epidemiol. 2013;48(2):183–93. 10.1007/s00127-012-0539-0. 22777395 10.1007/s00127-012-0539-0

[49] MacKinnon DP, Fairchild AJ, Fritz MS. Mediation analysis. Annu Rev Psychol. 2007;58:593–614. 10.1146/annurev.psych.58.110405.085542. 16968208 10.1146/annurev.psych.58.110405.085542 PMC2819368

[50] Makhmud A, Thornicroft G, Gronholm PC. Indirect social contact interventions to reduce mental health-related stigma in low- and middle-income countries: systematic review. Epidemiol Psychiatr Sci. 2022;31: e79. 10.1017/s2045796022000622. 36348492 10.1017/S2045796022000622 PMC9677443

[51] Marcus M, Yasamy MT, van Ommeren M, Chisholm D, Saxena S. Depression: a global public health concern. World Health Organization Paper on depression. 2012:6-8.

[52] McAlister FA, Straus SE, Sackett DL, Altman DG. Analysis and reporting of factorial trials: a systematic review. JAMA. 2003;289(19):2545–53. 10.1001/jama.289.19.2545. 12759326 10.1001/jama.289.19.2545

[53] McLaren T, Peter LJ, Tomczyk S, Muehlan H, Schomerus G, Schmidt S. The seeking mental health care model: prediction of help-seeking for depressive symptoms by stigma and mental illness representations. BMC Public Health. 2023;23(1):69. 10.1186/s12889-022-14937-5. 36627597 10.1186/s12889-022-14937-5 PMC9831378

[54] Mehta N, Clement S, Marcus E, Stona AC, Bezborodovs N, Evans-Lacko S, Palacios J, Docherty M, Barley E, Rose D, Koschorke M, Shidhaye R, Henderson C, Thornicroft G. Evidence for effective interventions to reduce mental health-related stigma and discrimination in the medium and long term: systematic review. Br J Psychiatry. 2015;207(5):377–84. 10.1192/bjp.bp.114.151944. 26527664 10.1192/bjp.bp.114.151944 PMC4629070

[55] Mental Health Innovation Network. WHO Special Initiative for Mental Health. 2023.

[56] Miret M, Ayuso-Mateos JL, Sanchez-Moreno J, Vieta E. Depressive disorders and suicide: epidemiology, risk factors, and burden. Neurosci Biobehav Rev. 2013;37(10 Pt 1):2372–4. 10.1016/j.neubiorev.2013.01.008. 23313644 10.1016/j.neubiorev.2013.01.008

[57] MoHP. National Mental Health Strategy and Action Plan-2077. Kathmandu Ministry of Health and Population. 2077.

[58] Moshki M, Baloochi Beydokhti T, Cheravi K. The effect of educational intervention on prevention of postpartum depression: an application of health locus of control. J Clin Nurs. 2014;23(15–16):2256–63. 10.1111/jocn.12505. 24329943 10.1111/jocn.12505

[59] Mugisha J, Hanlon C, Knizek BL, Ssebunnya J, Vancampfort D, Kinyanda E, Kigozi F. The experience of mental health service users in health system strengthening: lessons from Uganda. Int J Ment Health Syst. 2019;13: 60. 10.1186/s13033-019-0316-5. 31516548 10.1186/s13033-019-0316-5 PMC6728966

[60] Muthén LK, Muthén BO. *Mplus User*’*s Guide*. 6th ed. Los Angeles, CA: Muthén & Muthén; 2007.

[61] National Statistics Office. National Population and Housing Census 2021 Accessed through https://censusnepal.cbs.gov.np/results/literacy on 30 April 2023. 2023. Kathmandu, Nepal: Government of Nepal, Office of the Prime Minister and Council of Ministers.

[62] NHRC. Report of National Mental Health Survey 2020. Kathmandu Nepal Health Research Council, Government of Nepal. 2021.

[63] Patel V, Simon G, Chowdhary N, Kaaya S, Araya R. Packages of care for depression in low- and middle-income countries. PLoS Med. 2009;6(10): e1000159. 10.1371/journal.pmed.1000159. 19806179 10.1371/journal.pmed.1000159 PMC2747016

[64] Reddy MS. Depression: the disorder and the burden. Indian J Psychol Med. 2010;32(1):1–2. 10.4103/0253-7176.70510. 21799550 10.4103/0253-7176.70510 PMC3137804

[65] Rickwood DJ, Braithwaite VA. Social-psychological factors affecting help-seeking for emotional problems. Soc Sci Med. 1994;39(4):563–72. 10.1016/0277-9536(94)90099-x. 7973856 10.1016/0277-9536(94)90099-x

[66] Rickwood DJ, Deane FP, Wilson CJ. When and how do young people seek professional help for mental health problems? Med J Aust. 2007;187(S7):S35-39. 17908023 10.5694/j.1326-5377.2007.tb01334.x

[67] Saade S, Lamarche AP, Khalaf T, Makke S, Legg A. What barriers could impede access to mental health services for children and adolescents in Africa? A scoping review. BMC Health Serv Res. 2023;23(1):348. 10.1186/s12913-023-09294-x. 37024835 10.1186/s12913-023-09294-x PMC10080850

[68] Schnyder N, Panczak R, Groth N, Schultze-Lutter F. Association between mental health-related stigma and active help-seeking: systematic review and meta-analysis. Br J Psychiatry. 2017;210(4):261–8. 10.1192/bjp.bp.116.189464. 28153928 10.1192/bjp.bp.116.189464

[69] ten Have M, de Graaf R, Ormel J, Vilagut G, Kovess V, Alonso J. Are attitudes towards mental health help-seeking associated with service use? Results from the European study of epidemiology of mental disorders. Soc Psychiatry Psychiatr Epidemiol. 2010;45(2):153–63. 10.1007/s00127-009-0050-4. 19381427 10.1007/s00127-009-0050-4 PMC2820660

[70] Thornicroft G, Mehta N, Clement S, Evans-Lacko S, Doherty M, Rose D, Koschorke M, Shidhaye R, O’Reilly C, Henderson C. Evidence for effective interventions to reduce mental-health-related stigma and discrimination. Lancet. 2016;387(10023):1123–32. 10.1016/s0140-6736(15)00298-6. 26410341 10.1016/S0140-6736(15)00298-6

[71] Upadhaya N, Regmi U, Gurung D, Luitel NP, Petersen I, Jordans MJD, Komproe IH. Mental health and psychosocial support services in primary health care in Nepal: perceived facilitating factors, barriers and strategies for improvement. BMC Psychiatry. 2020;20(1):64. 10.1186/s12888-020-2476-x. 32054462 10.1186/s12888-020-2476-x PMC7020582

[72] Waldmann T, Staiger T, Oexle N, Rüsch N. Mental health literacy and help-seeking among unemployed people with mental health problems. J Ment Health. 2020;29(3):270–6. 10.1080/09638237.2019.1581342. 30862221 10.1080/09638237.2019.1581342

[73] Weissman MM, Olfson M. Depression in women: implications for health care research. Science. 1995;269(5225):799–801. 7638596 10.1126/science.7638596

[74] Whiteford HA, Degenhardt L, Rehm J, Baxter AJ, Ferrari AJ, Erskine HE, Charlson FJ, Norman RE, Flaxman AD, Burstein R, Murray CJL, Vos T, Johns N. Global burden of disease attributable to mental and substance use disorders: findings from the Global Burden of Disease Study 2010. Lancet. 2013;382(9904):1575–86. 23993280 10.1016/S0140-6736(13)61611-6

[75] WHO. mhGAP intervention guide for mental, neurological and substance use disorders in non-specialized health settings: mental health Gap Action Programme (mhGAP) – version 1.0. Geneva, Swizerland: WHO. 2010. 23741783

[76] WHO. Depression and other common mental disorders: global health estimates. Geneva: World Health Organization; 2017.

